# Identification of Apple Varieties Using a Multichannel Hyperspectral Imaging System

**DOI:** 10.3390/s20185120

**Published:** 2020-09-08

**Authors:** Yuping Huang, Yutu Yang, Ye Sun, Haiyan Zhou, Kunjie Chen

**Affiliations:** 1 College of Mechanical and Electronic Engineering, Nanjing Forestry University, Nanjing 210037, Jiangsu, China; h.y.p_2010@163.com (Y.H.); yangyutu@njfu.edu.cn (Y.Y.); zhouhaiyanzj@njfu.edu.cn (H.Z.); 2 College of Engineering, Nanjing Agricultural University, Nanjing 210031, Jiangsu, China; kunjiechen@njau.edu.cn

**Keywords:** apple, identification, spatially resolved spectra, multichannel hyperspectral imaging

## Abstract

This paper reports the nondestructive detection of apple varieties using a multichannel hyperspectral imaging system consisting of an illumination fiber and 30 detection fibers arranged at source–detector distances of 1.5–36 mm over the spectral range of 550–1650 nm. Spatially resolved (SR) spectra were obtained for 1500 apples, 500 each of three varieties from the same orchard to avoid environmental and geographical influences. Partial least squares discriminant analysis (PLSDA) models were developed for single SR spectra and spectral combinations to compare their performance of variety detection. To evaluate the effect of spectral range on variety detection, three types of spectra (i.e., visible region: 550–780 nm, near-infrared region: 780–1650 nm, full region: 550–1650 nm) were analyzed and compared. The results showed that the single SR spectra presented a different accuracy for apple variety classification, and the optimal SR spectra varied with spectral types. Spectral combinations had better accuracies for variety detection with best overall classifications of 99.4% for both spectral ranges in the NIR and full regions; however, the spectral combination could not improve the results over the optimal single SR spectra in the visible region. Moreover, the recognition of golden delicious (GD) was better than those of the other two varieties, with the best classification accuracy of 100% for three types of spectra. Overall, the multichannel hyperspectral imaging system provides more spatial-spectral information for the apples, and the results demonstrate that the technique gave excellent classifications, which suggests that the multichannel hyperspectral imaging system has potential for apple variety detection.

## 1. Introduction

Apple is one of the most popular and valuable fruits in the world due to its texture, flavor, and nutrition [1,2]. The quality of the apple fruit is normally defined by physical characteristics (i.e., color, size, and texture) and chemical compositions (such as soluble solid content, acidity, and vitamins), and these quality parameters are influenced by the variety of apple fruit [3,4]. Apple from different varieties has a specific firmness, crispness, nutritional composition, and taste, which could determine the consumers’ choice [5,6]. Besides, Rosend et al. [7] reported that apple variety turned out to be the main factor influencing the quality and aroma properties of apple cider. Thus, the variety discrimination of apple fruit is important in the modern markets with respect to consumers and producers alike. 

Conventional analytical methods for evaluating quality and classifying apples are usually physical and chemical analyses like deoxyribonucleic acid analysis for chemical composition tests [8], liquid chromatography analysis for sugar or acidity tests [9], Magness–Taylor test for firmness analysis [10], and potentiometric sensors called ISFETs for variety detection [11], which are destructive, time-consuming, and complex experimental procedures. Some nondestructive techniques for apple quality and variety detection have been developed to address the shortcomings of physical chemical inspections. Nuclear magnetic resonance (NMR) has been demonstrated to be capable of discriminating apple juice of different varieties [12]. Tiplica et al. [13] applied acoustic measurement for apple variety discrimination, and 18 key characteristic points of obtained frequency spectra for apple fruit were identified and used for classifying the fruit variety. However, since the non-homogeneities of the internal environment of the apple, such as the varied density in different positions, existence of the kernel and tissue, could influence the propagation of sound waves, resulting in the errors in the frequency spectra making inaccurate detection. Marrazzo et al. [14] tested the feasibility of apple cultivars detection for intact apple fruit and fruit juice using an electronic nose chemical sensor, and the results showed that PCA analysis could separate samples from juice on day 1, but for day 2, PCA analysis could not show good separation. Similarly, Rudnitskaya et al. [15] proposed an electronic tongue as an analytical means for apple variety detection. Three analytical techniques were compared for evaluating the performance of the measurement of variety using the electronic tongue, and the results found that the electronic tongue is sensitive to organic acids, while the other two methods are sensitive to both organic acids and sugars, which suggests that the electronic tongue still had some limitations in apple variety detection.

Over the years, imaging and spectroscopic techniques have been widely used for assessing or grading food and fruit quality due to the advantages of rapidity, no need for sample preparation, and nondestructive detection [16,17,18,19]. Nowadays, the techniques are also employed in variety detection. Dubey and Jalal [20] reported on an improved sum and difference histogram texture feature to identify the species and variety of fruit and vegetables based on HSV (Hue, Saturation, Value) images. Abundant literatures have demonstrated appropriate algorithms that could further improve the results [21,22,23,24]. Ronald and Evans [25] classified apple varieties using Naive Bayes algorithms, which could obtain higher accuracies than those of principal components analysis (PCA), fuzzy logic, and MLP-Neural. While images are good at the recognition of color and shape of the object, establishing the relationship with internal quality is too tough. Visible and near-infrared spectroscopy is one of the most commonly used nondestructive techniques to provide quantitative and qualitative information about samples as the measured light is related to the composition and structure of the object. Mid-infrared (MIR) and near-infrared (NIR) spectroscopy were compared to evaluate the performance of recognition of apple juice from four varieties, and the results showed that the overall classification accuracies were similar, but both techniques could not identify the Elstar samples correctly [26]. In addition, another study reported on the comparison of classification accuracies for the identification of apple variety using visible reflectance spectroscopy over the spectral range of 300–700 nm and RGB color information, where better classification results were obtained by the spectroscopic technique [27]. Moreover, the comparison of different spectral preprocessing methods and mathematical models for the identification of apple variety using visible and near-infrared (VIS/NIR) spectroscopy was also presented in previous studies [2,3,4], which suggest that VIS/NIR spectroscopy has potential for the detection of apple variety. The VIS/NIR spectroscopy acquired the light from a specific area of the samples, where it is possible for the compositions and structure of apple fruit to vary in different positions, so the VIS/NIR spectra are not adequate to reflect the characteristics of the fruit, which could result in inaccurate detection.

Spatially resolved spectroscopy (SRS) acquires the reflectance from objects at different spatial distances between the illumination and detectors, and it enables the obtaining of information from the sample at different depths as the transport path of photons generally forms a “banana-shape” in the tissue of the biological materials [28]. There are various configurations for SRS, including single-fiber-based, multi-fiber-based, and hyperspectral imaging-based, for measuring the optical absorption and scattering properties of food products [29,30,31]. Furthermore, several research studies also studied quality prediction of fruit using individual spatially resolved (SR) spectra, and the results showed that each SR spectra presented varied predictions, which means the predictions were influenced by source–detector distances [30,32,33]. However, few studies reported on qualitative analysis for fruit using individual SR spectra or spectral combination. Moreover, for apple variety detection, most studies ignored the geographical and environmental influences for spectra as the samples of different varieties were from different locations. 

Therefore, in this study, apple samples of three varieties were harvested in the same orchard to avoid geographical and environmental influences. The feasibility of a new developed multichannel hyperspectral imaging system based on SRS [34] for variety detection of apple was analyzed and discussed. It enables the obtaining of 30 SR spectra over a wavelength range of 550–1650 nm from samples at a source–detector distance of 1.5–36 mm. The specific objectives of this study were to: (1) Acquire SR spectra of apples for three varieties at a spectral range of 550–1650 nm; (2) analyze the relationship between quality attributes and varieties; (3) develop partial least squares (PLS) discriminant models based on single SR spectra and spectral combinations, and analyze and compare the classification results for apple variety detection.

## 2. Material and Methods

### 2.1. Samples

A total of 1500 apples, 500 each of the three varieties (i.e., Red Delicious, Red Roman, and Golden Delicious), were hand-picked from an experimental field at Michigan State University’s Horticultural Research and Teaching Center in Holt, Michigan, USA. All samples were first stored in the cooling room at a temperature of 4 ℃. Before the experiment was started, apple samples were kept at room temperature (about 24 ℃) for at least 16 h to make sure each of the whole sample had a consistent temperature. The surface of each apple sample was cleaned and inspected to ensure no external defects, and then classified for three varieties.

### 2.2. Spatially Resolved Spectra Acquisition

**Figure 1:**
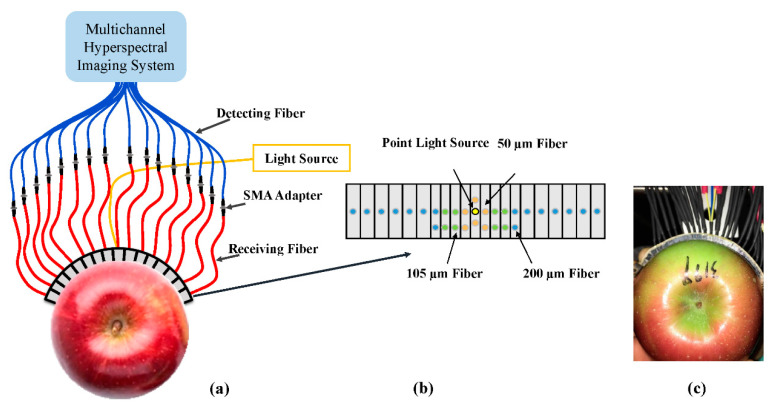
(**a**) Schematic of the multichannel hyperspectral imaging (MHI) system for acquisition of 30 spatially resolved reflectance spectra from a sample at light source–detector distances between 1.5 and 36 mm, (**b**) the arrangement of 30 fibers of three sizes (50, 105, and 200 µm) on the flexible probe, and (**c**) the experimental testing image.

Spatially resolved spectra for each apple sample were obtained using a new developed multichannel hyperspectral imaging system (Model 1003B-10152, Headwall Photonics, Inc., Fitchburg, MA, USA), which is assembled with an imaging spectrograph, a Vis-InGaAs camera covering the spectral range of 550–1650 nm with 229 variables, 35 200 µm detecting fibers, and associated optical hardware. Unlike conventional hyperspectral imaging systems that use an objective lens to focus the incoming light to the slit, the multichannel hyperspectral imaging system consists of 35 light-receiving fibers of 200 µm with a numerical aperture (NA) of 0.22. Figure 1 shows a schematic of the multichannel hyperspectral imaging system and the acquisition probe that consists of a 910 nm illumination fiber and 30 light-receiving fibers of three sizes (i.e., 50, 105, and 200 µm) arrayed in pairs with symmetry to the illumination fiber covering spatial distances of 1.5 to 36 mm. A detailed description of the multichannel hyperspectral imaging and acquisition probe is given in Huang, Lu, and Chen [34]. For the apple experiment, the multichannel hyperspectral imaging system was set with an integration time of 60 ms and the light source output was set for 240 W. During the measurement, the probe was in direct contact with the equatorial area of the apple sample to acquire 30 spatially resolved reflectance spectra. As the symmetric pair of spectra presented the same spatial distance between the light source and detectors, a total of 15 single SR spectra for different source–detector distances were obtained by averaging the symmetric spectra. The same experimental procedure was taken for gaining the spatially resolved spectra for a white cylindrical Teflon block of 50.5 mm height and 80 mm diameter as a white reference to calibrate the sample spectra. While the dark reference spectra were obtained by closing the light source in a dark room, the relative spatially resolved reflectance spectra were calculated using the following equation:

(1) $$R=\frac{R_{s}-R_{d}}{R_{w}-R_{d}}$$

where *R_s_* is the sample reflectance, *R_d_* is the dark reflectance, and *R_w_* is the white reflectance at the same source–detector distance. The relative reflectance spectra were used in further data analysis.

### 2.3. Quality Analysis of Apples

After spectral acquisition, surface color acquisition, firmness measurement, and soluble solid content (SSC) tests were performed on the apple samples. A digital camera was used for acquiring the skin color of apple fruit. Acoustic firmness of each of the samples was obtained by a desktop acoustic firmness sensor (AFS, AWETA, Nootdorp, The Netherlands), while another firmness parameter, the puncture maximum force, was subjected to by a Texture Analyzer (Model TA.XT2i, Stable Micro System, Inc., Surrey, UK). The SSC value for each of the apples was acquired from a handheld digital refractometer (model PR-101, Atago Co., Tokyo, Japan). ANOVA was performed on the quality parameters of three varieties using SPSS 18.0 statistics software. The level of P < 0.05 was considered significant in all analyses.

### 2.4. Apple Variety Classification Models

Partial least squares discriminant analysis (PLSDA) is a commonly used approach for statistical discrimination, and it was demonstrated that it could perform chemometric classification well [35]. PLSDA models were first developed for each of the 15 single SR spectra to determine the optimal single SR spectra for variety classification. Afterward, combinations of optimal single SR spectra and the remaining 14 single SR spectra were performed by cascading the variables of SR single spectra, which means the number of variables for two-SR was double. Likewise, three-SR spectra combinations were made by connecting the optimal two-SR spectra with each of the remaining 13 single SR spectra. The spectra combination procedure continued until the classification results could not further improve by adding additional single SR spectra. The spectral range over the visible, near-infrared, and full wavelength regions was analyzed and compared for apple variety classification.

The 1500 apples samples were randomly divided into two sets with 1000 samples for calibration and the remaining 500 samples for independent testing. Partial least squares discriminant analysis (PLSDA) models were developed using MATLAB R2017a (The MathWorks, Inc., Natick, MA, USA) coupled with PLS Toolbox 8.2 (Eigenvector Research, Inc., Wenatchee, WA, USA). Venetian blinds cross-validation is suitable for the relatively large samples that are already in random order [36]. It was used for the calibration set to decide the optimal number of latent variables based on the minimum classification error of cross-validation or too slight an improvement as the number of latent variables increased. The classification accuracy or recognition rate was applied to evaluate the performance of the models.

## 3. Results and Discussion

### 3.1. Differences of Quality Attributes for Apples of Three Varieties 

Table 1 shows the analysis of variance (ANOVA) for the quality parameters of apple fruit in three varieties using SPSS 18.0 statistics software. The level of P < 0.05 was considered significant in all analyses. Color is an important external property for apples. The apples in three varieties all had high values in the R channel, and they did not have a significant difference, which suggests that each apple contained an abundant red color. Red Delicious (RD) had small values in the G and B channels, which could explain why the peel of RD is red, while for Golden Delicious (GD), the values in the R and G channels were large, which indicated that the color for the peel of GD focused on the mixed colors of red and green, yielding a yellow color. For Red Roman (RR), the values decreased as the channel changed from R to G and to B, which suggested that the peel of RR could focus on a red color. For skin color analysis, the G channel presented a significant difference for apples in three varieties, while, in the B channel, RD and GD had a significant difference. Firmness and SSC are two main parameters of internal quality. The acoustic test shows the global properties of apple fruit, while the puncture measurement presents local firmness for apple fruit [19,33]. For internal quality parameter ANOVA analysis, SSC, acoustic firmness (AF), and puncture maximum force (PF) all had significant differences for apples in three varieties. In this study, GD had the highest SSC than the other two varieties, and RR showed the highest values for firmness of the two measurement methods. 

### 3.2. Spectral Characteristic Analysis

**Figure 2:**
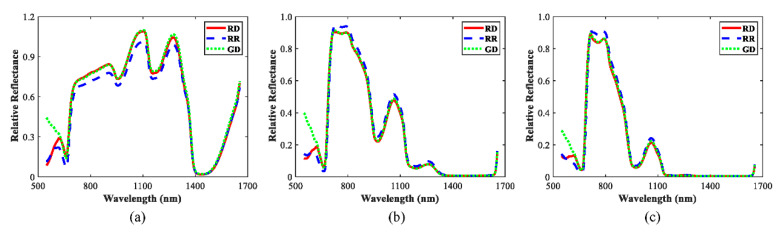
The mean relative spectra for apple fruit in three varieties obtained from (**a**) spatially resolved (SR) 1 of 50 μm fiber, (**b**) SR 4 of 105 μm fiber, and (**c**) SR 8 of 200 μm fiber covering spatial distances of 1.5, 6.0, and 12 mm.

**Figure 3:**
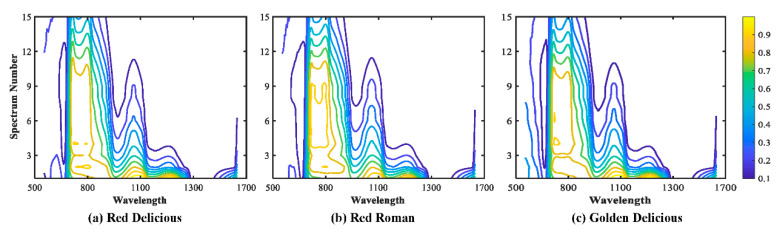
The contour maps for 15 SR spectra at each apple varieties over 550–1650 nm.

Figure 2 illustrates the mean relative spectra for apples in three varieties at source–detector distances of 1.5, 6.0, and 12.0 mm. The SR spectra for apples at three varieties overall had similar patterns except spectra around 550 nm, which could be due to the significance differences in the G channel at 546 nm for three varieties. It is a bit strange that GD had no noticeable difference at the chlorophyll absorption peak for 675 nm from the spectra with other two varieties, which could be because GD was one of the variety that had low chlorophyll content with yellow skin [37]. Figure 2 shows that the spectra for RR had some differences with other the two varieties over the spectral range around 750–1200 nm, and the possible explanation is that the firmness (AF and PF) of RR was quite a bit higher than those of the other two varieties (Table 1). Many studies reported that the near-infrared spectrum is closely related to some quality characteristics of fruits, such as firmness and soluble solid content. Structural differences could result in a difference in the spectrum over the near-infrared range. In the near-infrared (NIR) range, SR spectra were heavily influenced by the broad water absorption bands around 750, 970, and 1180 nm, and wavelengths beyond 1400 nm, which was in agreement with previous studies [32,33,36]. Moreover, there were distinct differences in the pattern of spectra for three SR spectra at different source–detector distances (i.e., 1.5, 6.0, and 12 mm) as they obtained the information of the samples at different tissue depths, which suggested that each SR spectra would present different classifications for the discrimination of three varieties in apple, and the combination of SR spectra should have more information on samples, resulting in better classification results. Figure 3 shows the contour maps for 15 SR spectra at three apple varieties. Prominent differences were observed from three contour maps for apple variety, especially at the spectral range of 550–900 nm. In addition, the intensity level decreased with the spectrum number, which is reasonable because, as the source–detector distances increased, the greater interaction of light with the sample tissue resulted in the attenuation of light intensity. Furthermore, each SR spectra at the wavelength range around or beyond 1300 nm had a very weak intensity level, which could be due to the water or O–H absorption bands. 

### 3.3. Discrimination Models for Apple Variety Detection

Table 2 summarizes the range, mean, and standard deviation for apple variety classification in the visible, near-infrared, and full wavelength range, respectively. For single SR spectra, the optimal SR spectra covering the visible range presented the best classification results of 0.976 for the discrimination of apple variety, which could be due to the fact that the variations in wavelength in the visible region were sensitive to color, and the apples in three varieties had significant differences in the G channel. Meanwhile, the signal-to-noise ratio (SNR) in the visible region was higher than that in the NIR region, which could also partly explain why the optimal single SR spectra in the visible region had a higher recognition rate than that in the NIR range. However, the range of single SR spectra covering the NIR region had more consistent classification results with a lower standard deviation than that in the visible region with 28.8% and 10.7% differences between optimal and worst classification results for the visible and NIR region, respectively. Each single SR spectra in the full wavelength range showed classification accuracies higher than 0.90 with a mean value of 0.942 for 15 single SR spectra for apple variety discrimination. Moreover, the differences between optimal and worst single SR spectra in the full wavelength range was only 7.3%. The optimal SR spectra in the visible region and full wavelength range were SR 1 and SR 2 with source–detector distances of 1.5 and 3.0 mm, respectively, which could provide more information about the top layer from samples. Besides, their classification accuracies were approximate, and these results indicated that the variables in the visible region for the optimal SR spectra at the full wavelength range made a higher contribution for model building; in other words, both of the two optimal single SR spectra were sensitive to skin color.

Spectral combination could not further improve the classification results for optimal SR spectra in the visible region, but the range of recognition rate for SR spectra in the visible region became better and consistent with a 7.5% improvement in mean value and 82.6% reduction in SD. The spectral combination for SR spectra in the NIR region made a prominent improvement for apple variety discrimination with a mean value of 0.982 and SD of 0.0068, representing a 12.9% and 72.7% improvement compared to single SR spectra, respectively. The optimal spectral combination for the NIR region comprised SR 14, SR 11, SR 8, and SR 10 with larger source–detector distances of 32, 20, 12, and 16 mm, respectively; these SR spectra were helpful for exploring deep layers of the samples. The interaction between the NIR light and the tissue of apples depends on the absorption and scattering properties, and the absorption is related to the chemical composition, while the scattering is related to the tissue structure. These SR spectra with large source–detector distances were selected as an optimal spectral combination in the NIR region, which indicate that the photons travelled a long distance and underwent multiple scattering events before being absorbed from the apple tissue, resulting in more information about chemical composition and structure being obtained. SSC is the typical chemical composition for apples, and firmness is one of the parameters on behalf of the structure for apple. As the analysis above, both SSC and firmness had significant differences for three apple varieties. These could explain why the spectral combination in the NIR region had better classification results. For spectral combination at the full wavelength range, SR 2 (3.0 mm source–detector distance), SR 14 (32.0 mm source–detector distance), SR 5 (9.6 mm source–detector distance), and SR 13 (28 mm source–detector distance) resulted in the best classification results of 0.994, representing only a 2.7% improvement over the optimal single SR spectra. The final classification results for both optimal spectra combinations in the NIR region and full wavelength were the same, and the averaged value and SD were also similar, which demonstrated again that the variables in the visible region could not provide any positive effect for spectral combination.

Table 3 summarizes the further breakdowns of classification results for three varieties by optimal single SR spectra and spectral combinations based on PLSDA models. In the visible region, the recognition rates for RD and GD in both training and test sets were close to 100%, whereas classification accuracies for RR were lower than 95% for both sets. The possible explanation is that the skin color of the RR is uneven, and some parts of the skin are a combination of red and green, so it is easy to affect the detection results. As shown in Table 3, there were 11 RR samples misjudged, 7 RR in the RD class and 4 in the GD class. In the NIR region, the identification rates in the training set were similar for three varieties of apples, and the maximum difference for recognition rates in the test set was only 4.5%. Besides, spectral combination resulted in consistently better test results than the optimal single SR spectra for each of the three varieties, with classification accuracies of 99.4%, 98.7%, and 100 %, which represented 8.2%, 11.7%, and 8.2% improvements, respectively. For the full wavelength range, the test results for GD were excellent with 100% classification accuracies for both optimal single SR spectra and spectral combination, while for RD and RR, the spectral combination improved the recognition rates for both training and test sets. Classification accuracies for GD were consistently higher than those of the other two varieties for both training and test sets for three types of SR spectra, while the RR had lower classification results, which could be due to the fact that RD and GD had a relatively single skin color, whereas the skin color for RR was composed of red and green. On the other hand, SSC as one of the chemical compositions for apples was directly influenced by light absorption, and signals at large source–detector distances had a greater impact on the measurement of the absorption. Although SSC for the three varieties were significantly different, the value of SSC for RR was in the middle between RD and GD, which could be possible to partly explain the lower classification results for RR.

Further analysis is shown in Table 4 to summarize two statistical parameters for assessing the performance of PLSDA models using optimal spectra for three types of spectrum based on different spectral ranges. Sensitivity is defined as the proportion of true positives that are correctly identified, while specificity is the proportion of true negatives that are correctly identified. In the visible region, both RD and GD had the highest value of sensitivity with the value of 1.000, whereas RR had a lower value of sensitivity; besides, the values of specificity for three apple varieties were similar. As discussed above, it could be due to the non-uniform surface color for RR, and visible light is sensitive to color, resulting in a lower value of sensitivity for RR. Both sensitivity and specificity displayed similar values in the NIR region for three apple varieties, which could be partly due to the fact that the surface color could have limited influence in the NIR region, and the spectra in the NIR region depended on the chemical composition and structural characteristics of apple fruit, while each quality parameter like SSC, AF, and PF had significant differences for three varieties, leading to relatively consistent results in the NIR region. For the full wavelength range, both sensitivity and specificity for GD showed the best results with the value of 1.000; moreover, the values of sensitivity and specificity for RD and RR were higher than 0.970, which suggested that the spectra in the full wavelength range, combined with the analysis advantages in the visible and NIR region, resulted in better overall results.

## 4. Conclusions

A multichannel hyperspectral imaging technique was applied to obtain 30 SR spectra from apples over 550–1650 nm for variety detection in this study. The SR spectra at three wavelength ranges were compared and analyzed to determine the influence of spectral range for variety assessment. Classification results based on PLSDA models were varied with each single SR spectra, showing 7.3%, 10.7%, and 28.8% differences between best and worst classification results for the visible, NIR, and full wavelength ranges, respectively. Spectral combination could further improve the recognition rates over the optimal single SR spectra for NIR and full wavelength ranges with excellent classification accuracies of 0.994; however, it was not effective for that in the visible region. The results also showed that better recognition rates were obtained for GD. The multichannel hyperspectral imaging system provide a new and effective means for apple variety detection.

## Figures and Tables

**Table 1:** Means and standard deviations of the quality parameters for apples with different varieties *.

Variety	R	G	B	SSC	AF	PF
RD	200 ± 17.5a	57 ± 8.9a	57 ± 11.1a	11.3 ± 1.0a	25.4 ± 8.3a	57.1 ± 11.3a
RR	202 ± 17.4a	123 ± 30.3b	73 ± 19.4ab	11.9 ± 1.0b	27.5 ± 2.7b	64.2 ± 13.6b
GD	195 ± 1.0a	212 ± 4.2c	85 ± 6.4b	12.6 ± 1.5c	21.6 ± 2.8c	50.3 ± 10.4c

* Numbers for the same columns with different letters are different at the level of 0.05 based on the analysis of variance; RD: Red Delicious; GD: Golden Delicious; RR: Red Roman; SSC: Soluble solid content; AF: Acoustic firmness; PF: Puncture maximum force.

**Table 2:** Ranges, means, and standard deviations (SD) of classification accuracies for recognition of three varieties of apples, by using partial least squares discriminant analysis (PLSDA) for the optimal SR spectra over the visible, near-infrared, and full wavelength range.

Spectra Type	Optimal Spectrum	Range	Mean	SD
Visible	Single (SR1)	0.758–0.976	0.883	0.0723
(550–780 nm)	Combination (SR1_2)	0.930–0.968	0.949	0.0126
NIR	Single (SR14)	0.822–0.910	0.870	0.0249
(780–1650 nm)	Combination (SR14_11_8_10)	0.970–0.994	0.982	0.0068
Full_wavelength	Single (SR2)	0.902–0.968	0.942	0.0178
(550–1650 nm)	Combination (SR2_14_5_13)	0.966–0.994	0.986	0.0068

**Table 3:** Classification results for three varieties of apples by using partial least squares discriminant analysis for optimal single spectrum and combination spectrum for three types of spectrum based on difference spectral ranges.

Spectral Type	Optimal Spectrum	Variety	Training Set/%	Test Set/%			
				RD	RR	GD	Accuracy
Visible (550–780 nm)	Single (SR1)	RD	99.4	172	7	0	99.4
		RR	94.2	1	144	0	92.9
		GD	99.7	0	4	172	100
NIR (780–1650 nm)	Single (SR14)	RD	94.5	159	11	6	91.9
		RR	95.7	7	137	7	88.4
		GD	96.0	7	7	159	92.4
	Combination (SR14_11_8_10)	RD	98.8	172	1	0	99.4
		RR	99.4	1	153	0	98.7
		GD	100	0	1	172	100
Full (550–1650 nm)	Single (SR2)	RD	96.0	167	9	0	96.5
		RR	92.8	6	145	0	93.5
		GD	99.7	0	1	172	100
	Combination (SR2_14_5_13)	RD	99.7	173	3	0	100
		RR	98.6	0	152	0	98.1
		GD	100	0	0	172	100

**Table 4:** Performance of the PLSDA models developed by optimal spectra for three types of spectrum based on difference spectral ranges.

Spectral Type	Sensitivity			Specificity		
	RD	RR	GD	RD	RR	GD
Visible (SR 1)	1.000	0.948	1.000	0.976	0.986	0.985
NIR (SR14_11_8_10)	0.988	0.981	0.994	0.985	0.974	0.985
Full (SR2_14_5_13)	0.994	0.981	1.000	0.976	0.988	1.00
